# Electrical Cell Impedance Sensing (ECIS): Feasibility of a Novel In Vitro Approach to Studying Venom Toxicity and Potential Therapeutics

**DOI:** 10.3390/toxins17040193

**Published:** 2025-04-11

**Authors:** Abhinandan Choudhury, Kaitlin Linne, Tommaso C. Bulfone, Tanvir Hossain, Abu Ali Ibn Sina, Philip L. Bickler, Bryan G. Fry, Matthew R. Lewin

**Affiliations:** 1 Adaptive Biotoxicology Lab, University of Queensland, St. Lucia, QLD 4072, Australia; abhinandan.choudhury@uq.edu.au; 2 Department of Emergency Medicine, University of California San Francisco Medical Center, San Francisco, CA 94143, USAtommaso.bulfone@ucsf.edu (T.C.B.); philip.bickler@ucsf.edu (P.L.B.); 3 Australian Institute for Bioengineering and Nanotechnology (AIBN), University of Queensland, St. Lucia, QLD 4072, Australiaa.sina@uq.edu.au (A.A.I.S.); 4 California Academy of Sciences, San Francisco, CA 94118, USA; 5 Ophirex, Inc., Corte Madera, CA 94925, USA

**Keywords:** snakebite envenoming, direct toxin inhibitors (DTIs), cytotoxicity, detachment, basement membrane, coagulotoxicity, electric cell-substrate impedance sensing (ECIS), marimastat, varespladib

## Abstract

Snakebite envenoming is often discussed in terms of lethality and limb loss, but local tissue injury and coagulotoxic effects of venom are significantly more common acute manifestations of snakebite envenoming (SBE). Local tissue injury and the hemorrhagic and coagulotoxic effects of venom are challenging to study in live animals and can be ethically fraught due to animal welfare concerns such that attention to the 3Rs of animal welfare motivates the development of in vitro techniques in this arena. Herein, we tested the use of a wound-healing study technique known as Electric Cell-Substrate Impedance Sensing (ECIS) to assess populations of cultured cells exposed to venom with or without sPLA_2_ and/or metalloprotease inhibitors (varespladib and marimastat, respectively). For comparison, the StarMax coagulation analyzer for coagulotoxicity was further used to evaluate the venoms and the neutralizing capabilities of the abovementioned direct toxin inhibitors (DTIs) against the same venoms examined using ECIS. Three viper and three elapid venoms that were examined for their effects on H1975 cells were *Agkistrodon contortrix* (Eastern Copperhead), *Crotalus helleri* (Southern Pacific Rattlesnake), and *Vipera ammodytes* (Horned Viper) and *Naja atra* (Chinese Cobra), *Naja mossambica* (Mozambique Spitting Cobra), and *Naja nigricollis* (Black-necked Spitting Cobra), respectively. The combination of cellular and coagulation techniques appears to usefully discriminate the in vitro capabilities and limitations of specific inhibitors to inhibit specific venom effects. This study suggests that ECIS with or without concomitant coagulation testing is a feasible method to generate reproducible, meaningful preclinical data and could be used with any type of cell line. Importantly, this approach is both quantitative and has the potential of reducing animal use and suffering during the evaluation of potential therapeutics. To further evaluate the potential of this method, rescue studies should be performed.

## 1. Introduction

Snakebite envenoming kills or significantly injures more than 500,000 people each year with a long-term injury (morbidity) prevalence of between 3 and 4:1 compared with fatalities [1,2,3]. Delays in care result in deaths and irreversible tissue injuries such that there remains an unmet need for out-of-hospital treatments to improve overall outcomes [4]. In addition, snakebites also affect both working, food, and companion animals, imposing significant burdens on veterinary resources and causing economic losses in rural communities due to livestock deaths [1,2,3,5]. Recognizing the severity of this global health gap in care, the World Health Organization (WHO) designated snake envenoming as a “Category A” Neglected Tropical Disease in 2017, based on the urgent need for stable, accessible, affordable, and effective antivenoms and field antidotes that can be distributed globally for both human and animal use [1,2].

While the prevention of lethality has long been the focus of therapeutic development for snakebites, the assessment of tissue damaging venom effects and treatment efficacy presents significant challenges that are particularly reliant on models [6]. In most cases, the test animals must be sacrificed, which causes distress not only to the animals but also to the researchers [7]. Moreover, conducting in vivo experiments is expensive and ever more highly regulated for all the above reasons [8]. These obstacles along with a lengthy procedure delay the progress of research and the development of effective treatment solutions [9]. Thus, there is a pressing need for more in vitro, potentially more affordable, and higher-throughput models for testing venoms, antidotes, and antivenoms in which tissue injury is the focus of investigation.

**Figure 1:**
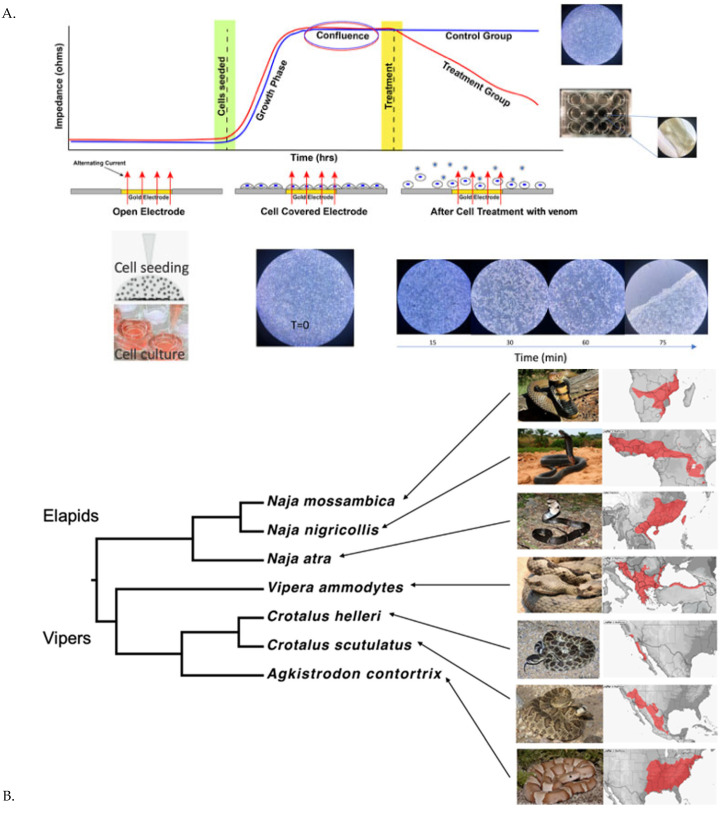
(**A**)**:** Showing schematic diagram representing the ECIS process adapted with permission from Applied Biophysics. Photomicrographs show the time course of *V. ammodytes* venom’s formation of resistance-dropping lacunes and dehiscence from a culture plate. (**B**): Showing the global distribution of the snake species used in this study.

To address this need, we tested the feasibility and efficacy of Electric Cell-Substrate Impedance Sensing (ECIS) as a novel method to quantitatively evaluate the effects of snake venoms and small-molecule direct toxin inhibitors (DTIs) to abrogate the cytotoxic effects of various venoms. ECIS has previously been employed in various cytotoxicity studies, including the testing of natural products, nanoparticles, and inhibitors to the Zika virus but, to our knowledge, not in the field of toxinology [10,11,12,13]. Briefly, cells are grown in specialized wells with electrodes at the bottom. As the cells grow to confluency, resistance increases, and this resistance can be monitored in real time through live traces displayed by the software; see Figure 1A. Resistance changes (gains or losses) indicate whether cells remain attached to the culture plate as well as leakage between cell–cell contacts [14]. We aimed to determine whether this technique has utility for elucidating how venoms affect cells and whether direct toxin inhibitors (DTIs) could preserve cellular attachments utilizing the ECIS system. To add an additional dimension to these evaluations, we used coagulation analysis of human plasma to examine the coagulotoxic properties of the same venom and inhibitor lots as previously described [15,16,17,18,19,20,21]. For this feasibility study, three viper and three elapid venoms were selected based on their reported potency to cause cytotoxicity and coagulotoxicity. The vipers chosen were *Agkistrodon contortrix* (Eastern Copperhead), *Crotalus helleri* (Southern Pacific Rattlesnake), and *Vipera ammodytes* (Horned Viper). The elapids selected were *Naja atra* (Chinese Cobra), *Naja mossambica* (Mozambique Spitting Cobra), and *Naja nigricollis* (Black-necked Spitting Cobra); see Figure 1B. These snakes are held accountable for substantial morbidity and mortality in America, Europe, Africa, and Asia due to their complex venom arsenal [22,23,24,25,26,27,28]. Among the myriad toxins present in these venoms, metalloproteinases (SVMPs) and phospholipase A_2_ (PLA_2_), particularly, play critical roles in cytotoxicity and coagulotoxicity and are appealingly important targets for synthetic enzyme inhibitors such as varespladib and marimastat [29,30,31,32,33].

SVMPs are instrumental in degrading extracellular matrix (ECM) proteins, disrupting basement membranes, and damaging endothelial cells, leading to significant tissue damage and bleeding [29,34,35]. PLA_2_ enzymes and related toxins, on the other hand, hydrolyze phospholipids in cell membranes, induce calcium influx, cause mitochondrial damage, and generate reactive oxygen species (ROS), collectively resulting in cell lysis, myotoxicity, and cell death [33,35,36,37]. In terms of coagulotoxicity, SVMPs can activate clotting factors in the coagulation cascade, such as the activation of Factor X by *Dabioa russelii* venom, which can lead to the formation of numerous microthrombi until clotting factors are depleted [29,38]. PLA_2_ can contribute to coagulotoxicity by binding to coagulation Factor Xa (FXa) and preventing the formation of the prothrombinase complex, as reported for *N. nigricollis* [30,39]. In both cases, these actions result in a net anticoagulant effect in human physiology. Together, these complex envenoming effects create temporally acute clinical challenges with long-term physical, psychological, and economic consequences [40,41,42,43]. Survivors often endure permanent disabilities, such as limb deformities or amputations, resulting in personal and economic hardship. Given these challenges, there is an urgent need for alternative interventions, therefore making the development of DTIs against snake venoms a recent focal point of research. DTIs, including snake venom metalloproteinase (SVMP) inhibitors like prinomastat and marimastat, and the phospholipase A_2_ (PLA_2_) inhibitor varespladib have shown promising results in both in vivo and in vitro studies such that they have been widely regarded as candidates for long-needed field treatments [44].

Thus, this study focused on the implementation of ECIS to evaluate the potential cytotoxicity of snake venoms and used the StarMax coagulation analyzer to assess the coagulotoxicity of venoms. Additionally, it aims to initiate the development and validation of ECIS as a reliable testing method for venom cytotoxicity.

## 2. Results

### 2.1. Cell Detachment Analysis—ECIS

**Figure 2:**
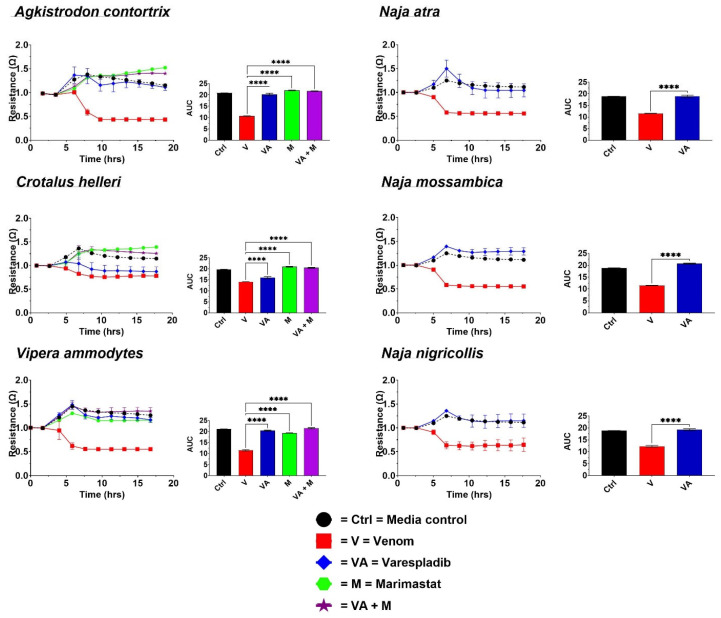
ECIS (Electric Cell-Substrate Impedance Sensing): For each venom (Left): line graphs of changes in impedance over time, with lower values relative to control indicating cell death; and (Right): values converted into AUC (area-under-the-curve) bar graphs, with lower AUC values indicative of cell death. Statistics are Brown–Forsythe and Welch ANOVA tests with post hoc Dunnett’s T3 multiple comparisons. The *p*-value classifications are as follows: * = *p* ≤ 0.05; ** = *p* ≤ 0.01; *** = *p* ≤ 0.001; **** = *p* ≤ 0.0001. All data are replicates (N = 3) ± standard deviation.

The treatment of cells with venoms resulted in a loss of resistance, indicating detachment from the plate substrate (Figure 2). The changes in resistance over time were then converted to area-under-the-curve (AUC) values for visualization. A higher AUC signifies the integrity of the cell confluence, suggesting health and survival, while a lower AUC indicates cell detachment or death (Figure 1B). All the venoms produced significant detachment (*p* < 0.0001) relative to negative control cultured cells (media-only cell growth).

For all the Viperidae venoms (*A. contortrix*, *C. helleri*, and *V. ammodytes*), the application of DTIs prevented cell detachment, with *p* < 0.0001. For *A. contortrix*, varespladib and the metalloprotease inhibitor marimastat both prevented detachment, suggesting that, for this species, cellular damage is induced by both toxin classes. However, a differential effect was evident for the other two viperid snake venoms. While for *C. helleri* only a marginal protective effect was produced by varespladib, a much stronger effect was evident for the metalloprotease inhibitor, suggesting that the metalloprotease toxins are the dominant toxin type contributing to the cytotoxic pathophysiological outcomes in the cell line used for this study. For *V. ammodytes*, while both toxin classes contributed to cellular detachment, evidenced by both inhibitor types having a significant effect (*p* = 0.0001), the neutralization of PLA_2_ toxins had a stronger effect in protecting cell membrane integrity relative to that of metalloprotease toxins (*p* = 0.0001), with the combination of varespladib and marimastat having the greatest effect.

In contrast to both metalloprotease and PLA_2_ inhibitors contributing to the protection from cellular detachment for the viperid snake venoms, only the PLA_2_ inhibitor varespladib had an effect protecting the integrity of the monolayer (Figure 2) for the elapid venoms. The lack of any inhibition by the metalloprotease inhibitor marimastat (data in Supplementary Materials File S1) is consistent with previous reports that metalloproteases in *Naja* venoms act in a non-cytotoxic manner; instead, it acts by cleaving fibrinogen to prevent clotting, hence contributing to venom-induced anticoagulation [45,46,47,48,49,50].

### 2.2. Coagulotoxicity Analysis—StarMax

**Figure 3:**
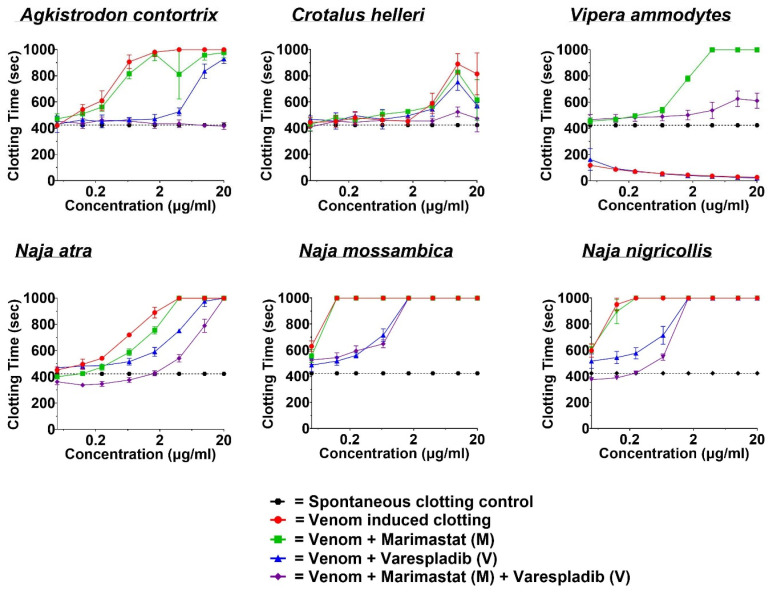
Line graph logarithmic views of 8-point venom dose–response curves (0.05, 0.125, 0.25, 0.66, 1.66, 4, 10, and 20 μg/mL) showing the effect upon plasma clotting as either anticoagulant (*A. contortrix*, *C. helleri*, all *Naja* species) or procoagulant (*V. ammodytes*) relative to the spontaneous clotting control of 423.1 ± 5.8 s. All data are replicates (N = 3) ± standard deviation.

The spontaneous clotting time (negative control) of human platelet-depleted plasma was recorded as 423.1 ± 5.8 s. There were no significant differences between the spontaneous clotting time and the DTI-treated controls: marimastat (M) recorded at 426.7 ± 27.4 s, varespladib (V) at 430.7 ± 32.1 s, and the combination of varespladib and marimastat (V + M) at 406.2 ± 27.5 s. The concentration–response effects of the venom serial dilutions were first recorded as line graphs, where clotting time to the spontaneous clotting time was either increased for anticoagulant venoms or decreased for procoagulant venoms (Figure 3). Overall, the combination of varespladib and marimastat was more consistently effective at normalizing coagulation than either one alone in the presence of venom-perturbed samples.

The viperid snake venoms were divided between anticoagulants (*A. contortrix* and *C. helleri*) and procoagulants (*V. ammodytes*). At the highest concentration of 20 µg/mL, the anticoagulant venoms extended the plasma clotting time to the machine maximum read of 999 s but with *A. contortrix* displaying greater potency than *C. helleri* by reaching this level at lower venom concentrations. While both varespladib and marimastat displayed moderate neutralization effects, the combination of the two inhibitors exhibited an extremely potent effect. This indicates that not only are PLA_2_ and metalloprotease toxins involved in the anticoagulation effects of *A. contortrix* and *C. helleri* venoms, but the two toxin types appear to have synergistic interactions (Figure 3). At the highest concentration of 20 µg/mL, the procoagulant *V. ammodytes* venom shortened the clotting time to 26.1 ± 0.4. The application of marimastat neutralized the procoagulant toxins but revealed low levels of background anticoagulant PLA_2_ toxins, a trait noted for venoms in the Palearctic clade previously [18]. The combination of the metalloprotease inhibitor marimastat combined with the PLA_2_ inhibitor varespladib restored the plasma clotting time to near spontaneous levels. Some of the residual anticoagulant activity observed could potentially be mitigated by using higher concentrations of varespladib in future studies. The highest degrees of anticoagulant activity were evident in all the *Naja* species. All were well neutralized by the combination of varespladib and marimastat, with varespladib alone having a greater effect than marimastat alone.

## 3. Discussion

Briefly, this study evaluated the feasibility of a novel application of the ECIS method to elucidate venom effects on the cell membrane integrity and pharmacological effects of DTIs on prevention of in-vitro venom-induced cellular detachment. In parallel, we analyzed the impact of the same venoms on coagulotoxicity for a comparison and contrast of the pharmacological effects of venom and inhibitors on different systems commonly affected by venoms. It is to be noted that the coagulation data provided a complementary understanding of the venom’s effect on coagulopathy, which plays a pivotal role in snakebite pathology. As both SVMPs and PLA_2_ are crucial mediators of cytotoxicity and coagulopathy, investigating both the parameters gives a better insight into the detailed assessment of DTI efficacy. For example, while both varespladib and marimastat prevented cellular detachment, only varespladib prevented the Agkistrodon anticoagulant effect, suggesting that sPLA2 blockade could be sufficient to mitigate these effects, while marimastat might only be effective at preventing cellular basement membrane detachment. All the venoms tested exhibited both cytotoxic and coagulotoxic properties, though with notable variations in their modes of action, whether in terms of cytotoxicity or coagulopathy. Overall, the findings suggest the potential of the ECIS technique to aid in the evaluation of the cytotoxic effects of venom in any chosen cell line. Successful results from this study could fulfill the conditions of the “four Rs”—Reduction, Replacement, Refinement, and Responsibility—in animal testing with a procedure that provides more relevant and reliable data while using human cells directly.

Both viperid and elapid venoms triggered cell death, as evidenced by the drop in electrical impedance. The efficacy of the DTIs varied across species (Figure 2). While all the DTIs were effective against *A. contortrix* and *V. ammodytes*, varespladib alone was less effective against *C. helleri* (B), where protection from cellular detachment relied more on marimastat and the combination of varespladib and marimastat. In contrast, varespladib alone was sufficient to mitigate the cytotoxic effects in elapids. Interestingly, the combination of varespladib and marimastat (V + M) proved to be the most effective in curbing coagulotoxicity across all venoms, followed by varespladib alone—except in the case of *V. ammodytes* (Figure 3). These results support the idea that combination therapy could offer greater protection against both the cytotoxic and coagulotoxic effects induced by these venoms than individual drugs. This highlights the potential of using in vitro techniques such as ECIS to test therapies for more comprehensive management of venom-induced toxicities sufficiently to replace at least some animal studies.

PLA_2_ and SVMPs are well known for their ability to damage cells and contribute to coagulopathy [29,30,33]. While we did not specifically analyze these venoms for their toxin content, the venom of *A. contortrix* has been reported to contain high levels of PLA_2_ (21.7% of type K49) and SVMPs (29.7% of type P-I) [51]. K49 PLA_2_s are basic and not dependent on Ca^2+^ for their activity but are more cytotoxic compared to D49-type acidic PLA_2_s [36]. K49 PLA_2_ in *A. contortrix* venom may explain the rapid cytotoxic effects observed even at lower venom concentrations than other tested venoms. Similarly, the high percentage of P-I type SVMPs, which are known to cause proteolysis, myonecrosis, and apoptosis, could contribute to the increased cytotoxic activity [29]. The anticoagulant activity observed in this study can be attributed to the presence of fibrolase, a P-I SVMP known for its fibrinolytic properties, and the presence of D49 type PLA_2_, which is known to have anticoagulant properties [29,30,33,51]. Previous studies have also reported the anticoagulant effects of *A. contortrix* venom, with the inhibition of FXa being a significant factor [52]. However, no significant difference in clotting time was observed between the venom-treated samples and the control in that study, which may be attributed to toxin variance due to the geographical location or source of the venom [53]. It was interesting to find that both DTIs, as well as their combination, completely neutralized the cytotoxic activity, indicating that, regardless of whether the toxicity is derived from SVMPs or PLA_2_s, the DTIs used effectively protected the cells. On the other hand, the combination drug provided protection against coagulotoxicity, followed by varespladib and then marimastat. This also suggests that the anticoagulation observed may be significantly influenced by the synergistic action of both SVMPs and PLA_2_s.

Among the two types of *Crotalus helleri* studied, we selected *C. helleri* (Type B) due to its known propensity for causing painful local tissue injury [54], attributed to the high concentration of SVMPs in its venom arsenal, while Type A sometimes has additionally neurotoxic clinical presentations [55,56,57,58]. In this instance, marimastat was able to preserve cell adhesion at control levels, while varespladib had little effect. Since marimastat is a known SVMP inhibitor, this suggests that the observed cellular detachment was primarily driven by SVMPs, which were effectively neutralized by the inhibitor. In a previous study, crotamine-like protein was detected in *C. helleri* venom, which induced necrosis by disrupting the cell membrane and endothelial walls [59]. The actions of hydrolysis by the SVMPs of such venoms were also reported in a separate study [57]. The action on fibrin is brought about by a fibrinolytic enzyme known as hellerase, which has been isolated from *C. helleri* [60]. It was interesting to see that the anticoagulant activity was not as strong as that of the other anticoagulant venoms used in that study. Yet, the anticoagulant activity appeared to be primarily influenced by PLA_2_, which is abundant in the venom though at a lower percentage [61], as varespladib, which targets PLA_2_, yielded better results in controlling coagulotoxicity than marimastat. Notably, the combination of both inhibitors, varespladib and marimastat, outperformed either drug alone, which supports the idea that both SVMPs and PLA_2_s act synergistically in *C. helleri* venom, contributing to its overall toxicity.

Among the venoms tested, *V. ammodytes* required the highest concentration of venom (25 µg/mL) to induce comparable cellular detachment, while it exhibited strong procoagulant activity even at the lowest venom concentration in plasma. This is consistent with the known characteristics of *V. ammodytes*, which is typically associated with coagulotoxic and neurotoxic effects, with necrosis being a rare occurrence [19,62,63,64,65]. These physiological effects are primarily due to the abundance of both PLA_2_, including the myotoxic sPLA_2_ homologue AtnL, and SVMPs of the P-II and P-III classes in the venom [65,66]. The application of either marimastat or varespladib successfully inhibited cellular detachment, suggesting that these toxins may act synergistically to induce cytotoxic effects but with metalloproteases contributing more strongly. Previous studies have reported the cytotoxicity of *V. ammodytes* venom on other cell lines, with IC_50_ values as low as 1.8 ± 0.1 µg/mL for CaCo-2 (human epithelial colorectal adenocarcinoma cells) [67]. Additionally, an isolated hemorrhagic P-III SVMP, VaH4, has been shown to cleave the extracellular matrix (ECM), reduce adhesion of HeLa cancer cells, and decrease cell viability [68]. The procoagulant activity of *V. ammodytes* venom, attributed to the presence of P-III SVMPs [29,69], aligns with our previous findings [19]. However, in the earlier study, the direct toxin inhibitors (DTIs) used were prinomastat and DMPS (2,3-dimercapto-1-propanesulfonic acid), with prinomastat demonstrating superior efficacy between the two [19]. In the study involving *D. russelii*, marimastat led to anticoagulant effects due to the neutralization of procoagulant metalloproteases but not background anticoagulant PLA_2_ toxins [18], which were mitigated by the application of varespladib in combination. In the current study, we explored the effects of marimastat and varespladib, and the combination of marimastat and varespladib successfully neutralized the venom’s effects [19]. It should be noted that the metalloprotease inhibitor prinomastat has shown some inhibitory potential with PLA_2_ toxins when studied in vitro, possibly suggesting a comparatively broader range of inhibition for some metalloprotease inhibitors and warranting further investigation[18]. 

Cobra (*Naja*) species are the most widely found venomous elapids found both in Asia and Africa and are most commonly notorious biters, which results in several physiological effects like neuromuscular paralysis, coagulopathy, and necrosis leading to death [70,71]. The three *Naja* species used in this work were previously reported to be highly cytotoxic, which matches with the implications of necrosis on the human bite victims [71]. In a comparative study between *N. atra* venoms from east and west Taiwan, it was found that this cobra’s venom consisted largely of the cytotoxins, followed by neurotoxins and then PLA_2_s [72], whereas no SVMP was reported [27,72,73]. *N. mossambica* and *N. nigricollis* were reported to have a similar prevalence of three-finger toxins followed by PLA_2_s, and both were equally cytotoxic [74]. While the venoms contain two classes of cytotoxins (three-finger toxins (3FTXs) and PLA_2_s), it has been shown previously that the PLA_2_ toxins potentiate the actions of the 3FTXs, and, therefore, the application of the PLA_2_ inhibitor varespladib is effective in blocking this pathophysiological effect [75]. With regard to coagulotoxicity, in alignment with earlier studies, *N. atra* on other hand was not as strongly anticoagulant as *N. mossambica* and *N. nigricollis* [76]. In a previous study in which *N. mossambica* and *N. nigricollis*, along with other African spitting cobras, were tested under similar experimental conditions, a significant drop in anticoagulation was observed with varespladib, indicating that varespladib could effectively inhibit PLA_2_-mediated Factor Xa inhibition [17]. From these observations, it can be hypothesized that the combination of varespladib and marimastat (V + M), followed by varespladib alone, might provide significant protection against venom-induced coagulotoxicity in *Naja* species, similar to the results observed with viper venoms. However, it was interesting to see that the combination drug was quite effective compared to either DTI alone, despite marimastat alone having minimal effects on this sample, indicating that the anticoagulation action was mainly brought about by the PLA_2_ present in the venom (Figure 2). Overall, the combination drug showed better efficacy for preventing cellular detachment and coagulotoxicity for the *Naja*s as with the vipers.

Marimastat and varespladib, as small-molecule enzyme inhibitors or DTIs, target the enzymatic sites of their respective enzymes [77]. Marimastat inhibits SVMPs by binding directly to the Zn^2^⁺ ions in the active site, preventing the enzyme from interacting with substrates like collagens or coagulation factors [78]. Varespladib, on the other hand, targets PLA_2_ by binding to the fatty acid substrate site, thereby inhibiting its interaction with the substrate and variously blocking its enzymatic activity or membrane docking site [79,80,81,82]. These mechanisms are crucial for reducing the cytotoxic and coagulotoxic effects of snake venoms. Marimastat has proven effective against venoms from *Echis* and *Crotalus atrox* in inhibiting cytotoxicity, while varespladib was surprisingly ineffective against any venom tested, including *N. nigricollis* [83]. However, when marimastat was combined with varespladib, there was a notable improvement in cell viability against *Bothrops asper* venom. Similarly, the combination of marimastat and varespladib significantly reduced lesion areas in mice as well as dermonecrosis by *Bothrops asper*, *Crotalus atrox*, and *Echis ocellatus*, demonstrating the enhanced protective effects of the combined treatment [83]. The whole venom of *C. atrox* and a group-I metalloprotease, CAMP-2, which was shown to exert cytotoxic, collagenolytic, fibrinogenolytic, and hemolytic activities, were both neutralized by the application of marimastat and batimastat [84]. Marimastat has been shown to reduce the venom potency of *Bitis arietans*, *Bothrops jararaca*, *Crotalus atrox*, *Calloselasma rhodostoma,* and *E. ocellatus* venoms at a low EC_50_ concentration [85]. Varespladib was reported to significantly reduce lesion area and dermonecrosis caused by *N. nigricollis* and *N. pallida* crude venom [75]. This DTI prevented and delayed neurotoxic effects on CD-1 mice caused by *Oxyuranus scutellatus*, *Bungarus multicinctus*, and *Crotalus durissus terrificus*; it also reversed neurotoxicity and rescued juvenile pigs from lethal doses of *Micrurus fulvius* (Eastern coral snake) venoms [86,87]. An extensive and in-depth analysis of varespladib as a potential therapeutic for snakebite envenoming was conducted in a review by Lewin et al. (2022), where they examined around 30 publications on successful preclinical tests of varespladib against a wide range of venomous and medically important snake species [81]. A summary of other DTIs’ efficacies had been well investigated in another review paper by Silvia and Chowdhury (2024) [88]. These findings corroborate with the results achieved in this study, where both varespladib and marimastat exhibited extensive inhibitory effects on venoms from both vipers and elapids.

ECIS has already been used to evaluate cytotoxicity in numerous studies [89]; its novel application in this study may prove to be a valuable tool for assessing venom cytotoxicity against cells from different lineages. ECIS offers a high-throughput and user-friendly experimental setup. While our study introduces a new approach to the evaluation process, several factors should be considered for future research. Expanding the venom samples to include a broader geographical range and testing various cell types, including primary human skeletal muscle cells, is crucial, as these are common targets of cytotoxic venoms. Additionally, determining the IC_50_ of venoms and the EC_50_ of DTIs will provide more precise data. Rescue experiments in which venoms are applied to cells followed by DTIs after a delay should also be conducted to simulate real-life scenarios and assess the effectiveness of DTIs in comparison to or in combination with venom-specific antivenoms and premixing of drugs and venom in this study are a limitation of its scope as was the use of only one cell line. Moreover, the device used in this study of the ECIS technique allows the user to create artificial wounds in the plate to simulate wounds and healing [13], which could be readily adapted to ECIS with venoms and the study of recovery in the presence or absence of therapeutics.

This study, to the best of our knowledge, is the first to demonstrate ECIS as a potentially versatile instrument for analyzing venom cytotoxicity. Furthermore, it highlights the efficacy of DTIs, particularly the combination of varespladib and marimastat, in mitigating both cytotoxicity or cellular detachment and coagulotoxicity. Overall, this research not only contributes valuable knowledge to the existing body of resources but also serves as an instrumental study in demonstrating the potential of DTIs in venom research.

## 4. Materials and Methods

### 4.1. Approvals

All the work was undertaken under animal ethics approval: (a) Australian Red Cross (44 Musk Street, Kelvin Grove, QLD 4059, Australia) research approval #16-04QLD-10 (1 March 2025); (b) University of Queensland Biosafety Approval #IBC/134B/SBS/2015 (20 September 2023); (c) Human Ethics Approval #2016000256 (9 May 2024). 

### 4.2. Stock Preparation

#### 4.2.1. Venoms

All the venom samples were obtained from Adaptive Biotoxicology Lab’s cryogenic collection or supplied by Ophirex, Inc., Corte Madera, CA 94925, USA. These venoms were obtained in lyophilized form and were reconstituted by adding 50% glycerol and deionized water to produce 2 mg/mL concentrated stock and stored at −20 °C until used for experiments. The venoms (location if known) were *Agkistrodon contortrix* (South Carolina, USA), *Crotalus. helleri* Type B (Mias Canyon, Beaumont, Riverside, CA, USA), *Naja atra* (NK), *N. mossambica* (South Africa), *N. nigricollis* (West Africa), and *Vipera ammodytes* (Balkans, Europe). A Thermo Fisher Scientific™ NanoDrop 2000 UV–Vis Spectrophotometer (Thermofisher, Sydney, Australia) was used to measure the concentration in triplicate at 280 nm wavelength. All the venom work was conducted under animal ethics approval and ethics committee approval. 

#### 4.2.2. Culturing Protocol for H1975 Cells

The H1975 cell line, epithelial lung carcinoma, has a high adherence property and a good growth rate, which are essential for reaching a rapid confluency of cells. Hence, this cell line was selected for the current study and procured from the American Type Culture Collection (ATCC). The H1975 cell line was cultured in RPMI 1640 media (Cat# 11875093, Thermo Fisher Scientific, Waltham, MA 02451, USA) supplemented with 10% fetal bovine serum (Lot # 2310B, Bovogen Biologicals, Keilor East, VIC, Australia) and 1% penicillin–streptomycin antibiotics. The cells were harvested following standard cell culture protocols. Mycoplasma testing was conducted at regular intervals (once a month) throughout the culturing period. All the cell culture work was performed under sterile conditions to prevent contamination. Pilot studies were originally conducted using Vero cells (shown in Figure 1).

#### 4.2.3. Plasma

The frozen pooled human plasma was thawed and aliquoted at 1.2 mL quantities, followed by flash-freezing in liquid nitrogen. The aliquots were stored at −80 °C until required for testing. These aliquots were defrosted at 37 °C in a Thermo Haake ARCTIC water bath right before experiments. Human plasma work was performed under University of Queensland Biosafety Approval #IBC/134B/SBS/2015 and Human Ethics Approval #2016000256.

#### 4.2.4. Direct Toxin Inhibitors (DTIs)

Two direct toxin inhibitors (DTIs) and a combination of both were used to determine their efficacy against the abovementioned venoms. Metalloprotease inhibitors were purchased from Sigma-Aldrich: the marimastat (2S,3R)-morpholinecarbohydroxamic acid hydrochloride) > 95% (HPLC) N4-[(1S)-2,2-dimethyl-1-[(methylamino)carbonyl] propyl]-N1,2- dihydroxy-3-(2-methylpropyl) butanediamide (catalogue # M2699) and the PLA_2_ inhibitor varespladib (LY315920-Na) were provided by Ophirex, Inc., (Corte Madera, CA, 94925, USA). Both the inhibitors arrived in powdered form; varespladib was dissolved in ddH_2_O, and marimastat was dissolved in 10% dimethyl sulfoxide (DMSO) and further diluted using deionized water to form 20 mM concentration of DTI solution main stocks, respectively.

### 4.3. Assay Conditions

#### 4.3.1. Cellular Detachment Effects Evaluation by Electric Cell-Substrate Impedance Sensing (ECIS)

A Z-Theta 96-well array station (Applied Biophysics, Troy, NY 12180, USA) was used to check the effect of the venoms and the efficacy of the DTIs in curbing the venoms’ effects on H1975 cells. A Hamilton Vantage automatic liquid handler (robot) was used to prepare the 96-well ECIS (Cat# 96W20idf PET) plate according to the producer’s requirements and to add reagents according to protocol. The ECIS station was setup inside a CO_2_ Incubator (Heracell™ VIOS 160i, Thermo Fisher) in which 38 °C in a humidified atmosphere containing 5% CO_2_ was maintained.

The ECIS plate was prepared automatically by the robot according to the ECIS manual for stabilization. Once stabilized, the plate was returned to the robot, and 200 µL of cell suspension was automatically added to each well. The cell counts and viability were assessed using a Cellometer (Nexcelom Bioscience LLC, Lawrence, MA, USA) before each experiment to ensure accurate seeding densities and high viability (>90%). Cells were seeded in 96-well ECIS arrays at a density of 3.6 × 10^4^ cells per well, and the plate was sealed with an adhesive, gas-permeable seal (Cat# AB-0718, Thermo Fisher). All these steps were performed under ambient sterile conditions and temperatures inside the robot. The plate was then loaded onto the ECIS station, and incubation was carried out for 16–18 h to attain confluence of the cells, indicated by stable resistance traces.

After the incubation, the venoms and DTIs were prepared to the desired concentrations and added automatically using the robot to the culture plate. Working stock concentrations of the DTIs were prepared by diluting 20 mM stock solutions of each DTI with RPMI, respectively, to 0.076 mM of varespladib (VA), 0.363 mM for marimastat (M), and 0.076 mM + 0.363 mM of varespladib and marimastat for the combination drug “VA + M”. Automatic dilution and distribution across the plate (three replicates of each condition covering all 96 wells) of the venoms or DTIs were implemented so that the final concentrations of venoms in each well were 3.125 µg/mL (*Agkistrodon contortrix*), 1.5 µg/mL (*Crotalus helleri* B), 25.0 µg/mL (*Vipera ammodytes*), and 12.5 µg/mL for all *Naja* species, while the final concentrations of DTIs were varespladib 0.024 mM (VA) marimastat (0.118 mM), and varespladib + marimastat (VA + M; 0.024 mM + 0.118 mM). The venom concentrations were set based on preliminary testing to ascertain relative effects, with the venoms proportionally reduced so that cellular detachment maximum effect occurred within the assay mid-point time period, thereby providing a wide window within which to observe the relative impact of the DTIs. The experiment was resumed by re-initiating data accumulation. The plate was incubated for a further 16–18 h before concluding the experiment. All the steps were performed under sterile conditions. Upon finishing the experiment, the raw ABP data were processed through the ECIS software (v1.4.18.0 PC). A higher normalized resistance is associated with cell health and survival, while a lower resistance correlates with cellular death, as represented in the line curves (Figure 1) [90].

#### 4.3.2. Coagulotoxic Effect Evaluation by STA-R Max^®^

To determine the effect of venoms on the plasma clotting time as well as the efficacy of the DTIs, the STA-R Max^®^ (Stago, Asnières sur Seine, France) coagulation analyzer was used. The venoms were manually diluted to 100 μg/mL (working stock) by adding OK Buffer (Stago catalogue #00360) from 2 mg/mL venom stock. Working stock was loaded into the analyzer, which automatically ran eight-point concentration curves with serial dilutions of 1, 1/2, 1/5, 1/12.5, 1/30, 1/80, 1/160, and 1/400 (the final reaction concentrations of the venoms were 20, 10, 4, 1.6, 0.67, 0.25, 0.125, and 0.05 μg/mL). Next, 50 μL venom stock was added to a cuvette, followed by the addition of 25 μL of OK buffer, 50 μL of 0.025 M calcium chloride (Stago catalogue # 00367), and 50 μL of phospholipid (Stago catalogue #00597), and then the mixture was incubated for 2 min at 37 °C followed by 75 μL of plasma added to the cuvette after incubation, and, eventually, the clotting time was recorded. The coagulation activator kaolin (Stago C⋅K Prest standard kit, Stago catalogue #00597) was used as a positive control, while 1:1 glycerol/deionized water was used as the negative control for both plasma and fibrinogen studies instead of venom.

The abovementioned eight-point concentration curves were repeated for DTI efficacy to neutralize the toxic effect of venom on plasma, where the 25 μL of OK buffer (added to the cuvette before incubation) was replaced with 25 μL of 2 mM (M) or 0.004 mM (V) or a combination of varespladib + marimastat “V + M” (VA: 0.004 mM + M: 2 mM), prepared from 20 mM stock of each DTI. The final concentration in the cuvette was 200 μM for marimastat or 0.4 μM for varespladib or 0.4 μM for varespladib + 200 μM for marimastat (V + M), respectively. All the data were run in triplicates.

### 4.4. Data Analyses

Data plotting and statistical analysis were performed using GraphPad PRISM 10.0.0 (GraphPad Prism, Inc., La Jolla, CA, USA). The data points generated from the ECIS method and the coagulation analyzer were plotted to generate respective graphs. To determine the DTI’s efficacy against venom, the area under the curve (AUC) for both venom and venom + DTI was calculated using the software and plotted for the ECIS results.

## Data Availability

The original contributions presented in this study are included in the article. Further inquiries can be directed to the corresponding authors.

## Supplementary Materials

The following supporting information can be downloaded at: https://www.mdpi.com/article/10.3390/toxins17040193/s1, File S1: ECIS raw data and controls.
